# Fetal magnetic resonance imaging contributes to the diagnosis and treatment of meconium peritonitis

**DOI:** 10.1186/s12880-020-00453-8

**Published:** 2020-05-24

**Authors:** Yuanting Lu, Bin Ai, Weijuan Zhang, Hongsheng Liu

**Affiliations:** 1 Department of Radiology, Guangzhou Women and Children’s Medical Center, Guangzhou Medical University, 318 Renmin Middle Road, Guangzhou, 510623 Guangdong Province China; 2 Department of Ultrasound, Guangzhou Women and Children’s Medical Center, Guangzhou Medical University, Guangzhou, China

**Keywords:** Fetal MRI, Fetal ultrasound, Meconium peritonitis, Fetal ascites, Gathering bowel loops

## Abstract

**Background:**

Meconium peritonitis (MP) is a rare fetal disease that needs to be urgently identified for surgical intervention. We report a series of 35 patients diagnosed prenatally with MP by magnetic resonance imaging (MRI), illustrate the imaging findings and investigate the predictive value of these findings for postpartum management.

**Method:**

A consecutive cohort of patients diagnosed with MP who were born at our institution from 2013 to 2018 was enrolled retrospectively. The prenatal ultrasound and MRI findings were analyzed. Fisher’s exact probability test was used to evaluate the predictive value of MRI for surgical intervention between the operative group and the nonoperative group.

**Results:**

Ascites (30/35) and distended bowel loops (27/35) were two of the most common prenatal MP-related findings on fetal MRI. Of the 35 infants, 26 received surgical intervention. All fetuses with MRI scans showing bowel dilatation (14/26, *p* = 0.048) and micro-colorectum (13/26, *p* = 0.013) required surgery. There were no significant differences in the number of fetuses with meconium pseudocysts and peritoneal calcifications between the two groups.

**Conclusion:**

Fetuses with bowel dilatation and micro-colorectum on MRI may need postpartum surgical intervention. Infants with only a small amount of ascites and slight bowel distention were likely to receive conservative treatment.

## Background

Meconium peritonitis (MP) is a type of aseptic chemical peritonitis caused by the leakage of meconium from small bowel perforations in fetuses. MP has a low incidence of 1 in 30,000 live births [1] and was a fatal disease with a mortality rate of 80–90% in the early 1950s [2]. MP is usually caused by the imperforation or/and perforation of the intestinal tract, but the inherent pathogenic mechanism remains unclear [3–5]. Because of the compensatory function of the maternal placenta, fetuses show few or even no abnormalities when MP appears. It is difficult to diagnose infants with MP without prenatal imaging. The fatality rate remains over 40% according to the report by Shyu M. K et al in 2003 [6]. Identifying and diagnosing MP with an effective method may provide some clues for postpartum management in an earlier period.

Ultrasound is the most commonly used method for prenatal examinations, but sometimes the results are disturbed by many factors [7]. In recent years, the development of fetal MRI technology has been suggested to be safe and feasible. He et al. reported that fetal MRI could be used to define MP features in 8 cases [8]. He et al summarized the MRI findings of MP, including massive meconium ascites, meconium pseudocysts, dilated bowel loops and micro-colorectum. However, the limited number of reported cases was not convincing, and the value of fetal MRI in predicting the need for surgical intervention among infants with MP has not yet been clarified.

The purpose of this study was to summarize the MRI findings for identifying MP and to investigate the value of these findings for predicting the need for postpartum surgical intervention. We collected all patients diagnosed with MP who underwent fetal MRI at the Guangzhou Women and Children’s Medical Center from January 1st, 2013, to January 1st, 2018. Then, the patient characteristics, ultrasound and MRI findings, postnatal management strategies and outcomes were analyzed.

## Methods

Infants diagnosed with MP who were born at our institution from January 1st, 2013, to January 1st, 2018 were analyzed retrospectively. A total of 35 infants who received fetal MRI scanning were finally included in this research. The fetal MRI and medical histories of the fetuses and mothers were systematically collected, and a follow-up (1 year at least) was performed. All parents provided written informed consent for fetal MRI before scanning. All of the information involving the private messages of these patients is protected. Written informed consent was waived by our institutional ethics committee review board for this retrospective case series.

### Ultrasound imaging

In our hospital, routine prenatal two-dimensional (2D) transabdominal ultrasound scans were performed by an obstetric specialist in prenatal ultrasonography diagnosis using a color Doppler ultrasonography (Volusion E8, General Electric, USA; HD11, Philips, Netherlands). The obstetric specialist who diagnosed the infants based on fetal US had no conflicts of interest in the study.

### Magnetic resonance imaging

Noncontrast enhanced fetal MRI was performed on a 1.5-Tesla (Achieva, Philips, the Netherlands) or 3.0-Tesla (Skyra, Siemens, Germany) superconducting MRI scanner with a four-channel phased-array abdominal coil. Sedation was not used for either the fetus or mother. Our fetal MRI protocol included the following principles and sequences to scan the fetus: (1) All pregnant women received an MRI scan in the supine position in the morning and were told not to eat for at least 3 h and not to drink for at least 2 h so that the fetus would be less active, and the MRI examination was performed during this period. (2) The 1.5-T scanning parameters were as follows: axial T1-weighted turbo spin echo (TR: 200 ms, TE: 5 ms, slices: 3 mm) and coronal, axial and sagittal T2-weighted sense-body fetal turbo spin echo (TR: 15000 ms, TE: 120 ms, slices: 6 mm). On occasion, fat-suppressed T2-weighted spectral presaturation attenuated inversion recovery (SPAIR) echo sequences (TR: 598 ms, TE: 80 ms, slices: 3 mm) and balanced fast field echo sequences (TR: 3.5 ms, TE: 1.74 ms, slices: 4 mm) were preferred. (3) The 3.0-T scanning parameters were as follows: axial T1-weighted turbo spin echo (TR: 4.0 ms, TE: 1.8 ms, slices: 3 mm) and coronal, axial and sagittal T2-weighted sense-body fetal turbo spin echo (TR: 2000 ms, TE: 87 ms, slices: 6 mm) were performed. On occasion, T1-weighted imaging vibe fat-suppressed (TR: 4.2 ms, TE: 2.0 ms, slices: 3 mm) sequences were preferred. Sixteen patients underwent imaging with a 1.5-T scanner, and 19 patients underwent imaging with a 3.0-T scanner (since June 2015). Serial ultrasound examinations were performed to monitor disease progression after the MRI diagnosis.

The images were independently reviewed by two experienced fetal MRI radiologists, both with 15 years of experience and who were blinded to the postnatal outcomes. A third radiologist was included when disagreements about the diagnosis existed. The fetal MRI report consisted of abnormal findings, including seroperitoneum and total abdominal tube morphology, and the presence of amniotic fluid. The fetal outcomes were identified by medical records and autopsy.

### Follow-up and statistical analysis

All infants were followed up for at least 1 year. Descriptive statistics were used to analyze the number of fetal abnormalities in the study cohort. Fisher’s exact probability test was used to evaluate the surgical predictive value of MRI between the operation group and observation group in a 2 × 2 table. The MRI findings were also reported using a descriptive approach.

## Results

Thirty-five infants diagnosed with MP who underwent MRI scanning were collected and analyzed in our study. The median gestational age was 31 weeks (range, 24–39 weeks) at the time of MRI scanning. There was no family history of MP in any mothers in our cohort. The median maternal age was 28 years old (19 to 34 years old). Of all mothers, nine mothers had hepatitis B virus (HBV), and nine had mild alpha thalassemia. Nine developed intrahepatic cholestasis of pregnancy, including eight mothers with HBV and one with alpha thalassemia.

**Fig. 1:**
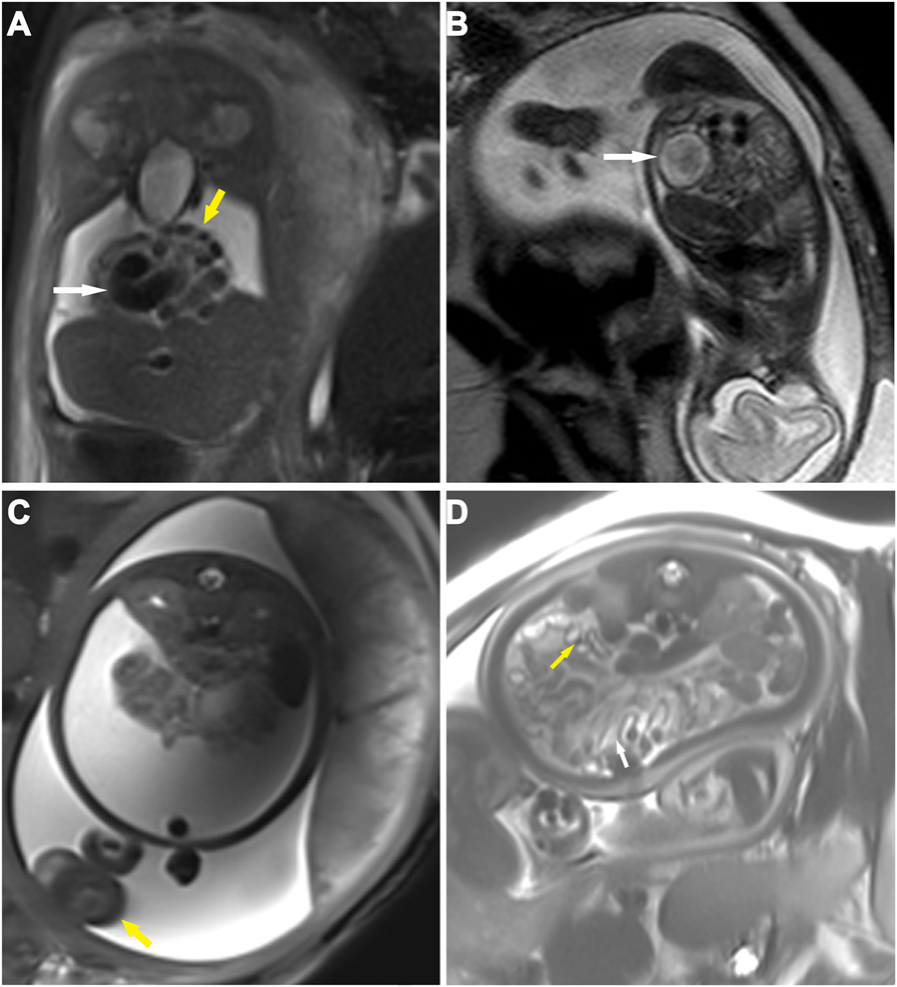
Fetal MRI findings of meconium peritonitis. **a** A coronal 3.0-T fat-suppression T2-weighted image (HASTE, TR = 15,000 ms, TE = 91 ms) reveals a moderate amount of ascites (a low-intensity signal) in the peritoneal cavity, compacted bowel loops (yellow arrow) and dilated bowels (white arrow) in a 30-week-old fetus with meconium peritonitis (MP). **b** A sagittal 1.5-T fetal T2-weighted image (TR = 15,000 ms, TE = 120 ms) shows mild ascites among bowel loops and a cyst (a 3.2 × 2.4 × 3.2 cm round foci with mix signal inside, white arrow) in a 24-week-old fetus with MP. **c** A transverse 3.0-T HASTE-FS image (TR = 15,000 ms, TE = 91 ms) indicates a high mount of ascites, gathered bowel loops (white arrow) and enlargement of the left scrotum (yellow arrow) in a 37-week-old fetus with MP. **d** A transverse 3.0-T fat-suppression T2-weighted image (HASTE, TR = 15,000 ms, TE = 91 ms) indicates gathered bowel loops (white arrow) and calcification spots (multiple small focuses or small nodules with low T2-weighted imaging signal, yellow arrow) in a 38-week-old fetus with MP

**Fig. 2:**
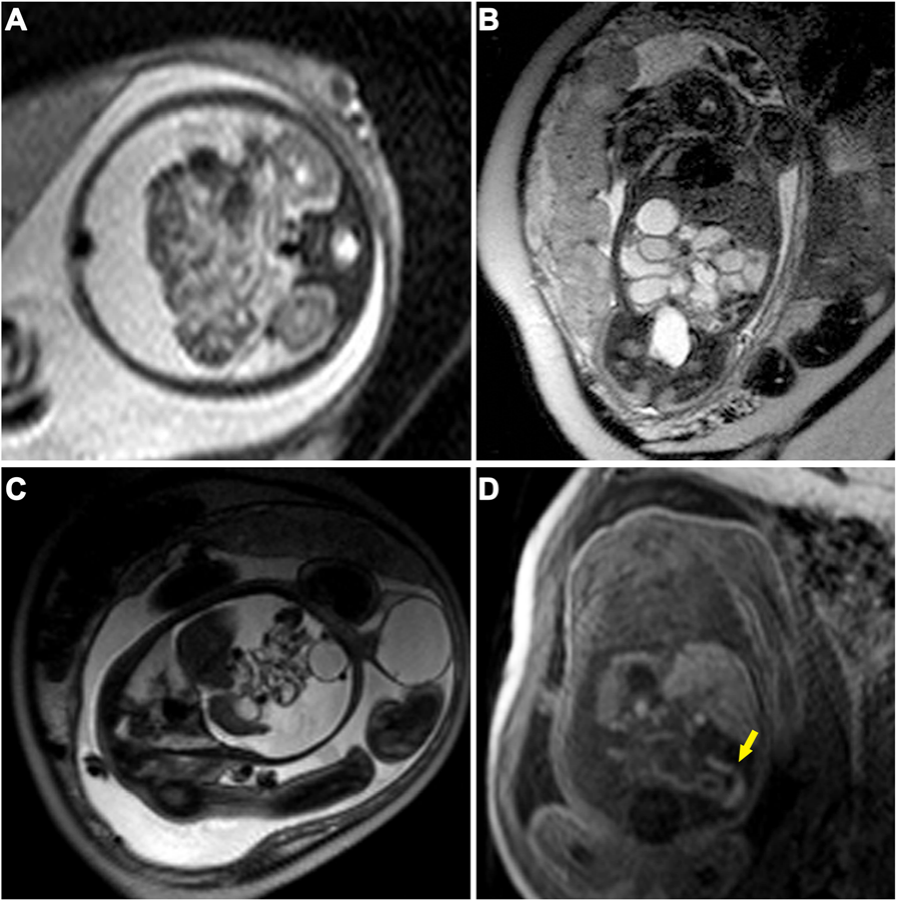
Fetal MRI findings of meconium peritonitis in two fetuses receiving surgery. **a**-**b** A 30-week-old fetus with MP. A transverse 1.5-T T2-weighted image (TR = 15,000 ms, TE = 120 ms) indicates a large amount of ascites and gathering bowel loops (**a**) and dilated bowels (**b**). C-D. A 35-week-old fetus with MP. A sagittal 1.5-T T2-weighted image (**c**, TR = 15,000 ms, TE = 120 ms) reveals a high amount of free ascites in the peritoneal cavity, gathering and distortion of poor filled small intestines, and significant swelling in the left scrotum. A coronal T1-weighted image (**d**, TR = 234 ms, TE = 4.6 ms) shows distal micro-colorectum (yellow arrow)

The imaging findings on both ultrasound and MRI are summarized in Table 1, and representative imaging findings are shown in Fig. 1 (ascites, gathered bowel loops, bowel dilatation, meconium pseudocysts, peritoneal calcification and hydroceles) and Fig. 2d (micro-colorectum). Ascites and bowel dilatation were two of the most common findings on both ultrasound and MRI. Distended bowel loops (27:4 on MRI or ultrasound), micro-colorectum (13:1), meconium pseudocysts (10:3) and hydroceles (13:4) were more likely to be depicted by MRI than by ultrasound. Of all 35 patients, 26 received surgical management. The intraoperative findings corresponded to the findings on MRI scanning. Atresia and perforation, two of the main causes of MP, were visualized in 22 and 12 infants, respectively.

**Table 1:** Prenatal findings of ultrasound and fetal MRI

Imaging findings^a^	Ultrasound	Fetal MRI
Ascites	26 (20)^b^	30 (20)
Gathering bowel loops	4 (20)	27 (20)
Bowel dilatation	12 (14)	15 (14)
Micro-colorectum	1 (14)	13 (14)
Meconium pseudocyst	2 (6)	7 (6)
Peritoneal calcification	3 (10)	12 (10)
Hydroceles	4 (8)	13 (8)

^a^ All 35 infants received both ultrasound and fetal MRI prenatal examinations, and the frequencies of MP-associated imaging findings are summarized

^b^ Among 26 infants receiving operation, imaging features were confirmed during operation as shown in the brackets

**Fig. 3:**
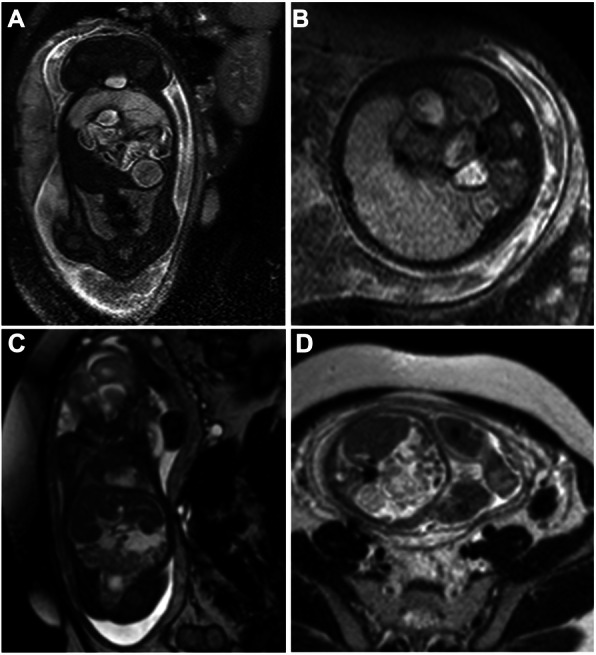
Fetal MRI findings of meconium peritonitis in two fetuses with existing cysts. **a**-**b**. A 34-week-old fetus received surgical intervention. A coronal T2-weighted image (**a**, SPAIR) reveals a cystic lesion in the left upper abdomen measuring 2.8 × 2.9 × 1.9 cm in size with a distinct thin border, linear-like incomplete separation and slightly high T2 signal inside. A transverse 1.5-T fetal T2-weighted image (**b**, TE = 15,000 ms, TE = 120 ms) shows that the bowel loops were pressed and shifted backward and dilated significantly with a diameter of 2.6 cm. **c**-**d**. A 27-week-old fetus received conservative treatment. A coronal T2-weighted image (**c**, SPAIR) shows a pseudocyst measuring 3.3 × 1.3 × 1.6 cm in size with irregular shape and high and slightly low T2 signal mixed inside. A transverse T2-weighted image (**d**, TR = 15,000 ms, TE = 120 ms) reveals a mild mount of ascites among bowel loops

To investigate the value of fetal MRI findings for predicting the need for surgical intervention, we divided all infants into an operative group and an observation group according to whether they received a postpartum operation, and we performed Fisher’s exact probability tests to analyze the correlations (Table 2). The presence of bowel dilatation (14/26, *p* = 0.048) and micro-colorectum (13/26, *p* = 0.013) seemed to be associated with the need for postpartum surgical intervention (Fig. 2). Of the 9 infants with nonsurgical interventions after delivery, 8 infants had ascites or/and distended bowel loops. There was only one infant with MRI signs of peritoneal calcifications or meconium pseudocysts who received conservative treatment (Fig. 3). Although the number of infants with meconium pseudocysts or peritoneal calcifications was not significantly different between the two groups, surgical intervention was considered when the MRI also revealed the presence of other findings other than ascites or distended bowel loops. Infants with hydrops in an enlarged scrotum did not need to undergo surgical treatment because of the self-healing capabilities of the body.

**Table 2:** Fetal MRI findings between operation group and observation group

Imaging features on MRI	Operation group (*n* = 26)	Observation group (*n* = 9)	*p* value^a^
Ascites	22	8	ns
Gathering bowel loops	19	8	ns
Bowel dilatation	14	1	0.048
Micro-colorectum	13	0	0.013
Meconium pseudocyst	6	1	ns
Peritoneal calcification	11	1	ns
Hydroceles	10	3	ns

^a^ Fisher’s exact probability test was used to evaluate the surgical predictive value of MRI between operation group and observation group. ns: *p* ≥ 0.05

In 26/35 (74.3%) cases, the parents elected for exploratory laparotomies at a median age of 1 day old (range, 0–9 days). Intestinal atresia (*n* = 14), perforation (*n* = 4) or both (*n* = 8) that occurred intraoperatively caused the development of MP. Most infants (*n* = 24) underwent resection and anastomosis, and the remaining infants (n = 2) underwent simple repairs. There were 3 cases of mortality (8.6%), including one intraoperative death and two postoperative deaths. After a one-year follow-up, 9 infants managed with expectant treatment and 23 managed with surgical intervention recovered well. In addition, 13 infants were confirmed to have hydroceles, but all recovered 6 months after birth.

## Discussion

MP, initially described in the English literature as early as 1838 [9], is a kind of chemical peritonitis caused by fetal autologous meconium. In our present study, we collected 35 cases of MP at our Women and Children’s Center from 2013 to 2018. The characteristics of the pregnancies and fetuses with MP and the ultrasound and MRI findings were systemically analyzed. Then, we retraced the treatments, pathological reports and outcomes of the fetuses with MP. In our current study, we found that fetal MRI could visualize more MP findings of intestinal perforation and atresia than other imaging methods, and the findings of bowel dilatation and micro-colorectum on MRI were two predictive factors for the need for surgery.

Similar to previous studies [1, 5, 10], the common characteristics of MP included fetal ascites, distended bowel loops, bowel dilatation, micro-colorectum, hydroceles, meconium pseudocysts and peritoneal calcifications in our current analysis. In 1983, Smith et al first reported that MRI could be used for scanning during pregnancy [11]. In the next decades, the development of rapid MRI technology has solved the disadvantage of long scanning times. Half-Fourier acquisition single-shot turbo spin-echo (HASTE) and true fast imaging with steady-state precession (true FISP) are two common sequences used for fetal MRI scanning and could acquire high-quality fetal images without the need for sedation or breath holding for 1 s. Early in 2002, Ertl-Wagner et al noted that fetal MRI was safe for both mothers during pregnancy and fetuses [12]. The American Food and Drug Administration suggested that fetal MRI could be used for scanning 3 months after conception. In the last 10 years, fetal MRI technology has been implemented and rapidly developed in China. However, systematic research on MP was lacking in Asian populations.

Fetal ascites was the most common antenatal finding of MP with both ultrasound (26/35, 74.3%) and MRI scanning (30/35, 85.7%) in our cohort, which is similar to the findings reported by Ping et al [13]. Sometimes, only fetal ascites appeared, and differential diagnoses such as fetal edematous syndrome (also called Bart’s syndrome) should be considered. Fetal pleural effusion, pericardial effusion and/or skin hydroncus can also be observed in addition to fetal ascites if Bart’s syndrome is present. Distended bowel loops was the second most common finding of MP on MRI. This symptom may be caused by fibrinous exudate and adhesions. A small pseudocyst does not influence the function of the digestive tract. In some cases, a large meconium pseudocyst required postnatal surgical treatment [6]. Infants who only had ascites and mild bowel loop distention were more likely to receive conservative treatment than to undergo surgery. It is speculated that the unexplained bowel perforation had been sealed off. Hydroceles and polyhydramnios were the third most common findings of MP on MRI in our study. When the ascites moved from the abdominal cavity into the scrotum, hydroceles developed. Fetal hydroceles could gradually be absorbed postnatally. Intestinal obstructions result in a decrease in swallowed amniotic fluid, which causes an increase in amniotic fluid in the uterus.

Dilated bowel, micro-colorectum and peritoneal calcifications were some of the other findings of MP. Bowel atresia could cause dilation of the proximal bowel. Nyberg et al suggested that a bowel internal diameter over 7 mm and a length over 15 mm should be diagnosed as intestinal dilation [14]. For the distal colon, the diameter reduces in size because of disuse atrophy. Then, the atretic intestinal tract appears perforated, and the meconium can enter into the abdominal cavity. In some cases, the location of the bowel perforation was difficult to distinguish. A perforation alleviates the pressure of the dilated bowel, which may lead to remission of the dilation of the proximal bowel. In our study, only 4 fetuses had symptoms of bowel dilation. The peritoneal calcifications formed because of the calcium salt particles of the meconium in the abdominal cavity reacted with the peritoneal inflammatory exudate. Shyu et al. reported that peritoneal calcifications on prenatal ultrasound were the most common finding of MP [6]. In our study, fetal ascites and distended bowel loops were the most common findings of MP on both ultrasound and MRI. The existence of ascites and/or distended bowel loops can interfere with the sonographers’ observation of other abnormities, while fetal MRI can provide more information to help determine the postpartum management strategy.

Surgery is the main method for the postnatal management for MP. Recent studies have achieved a better survival rate than that reported in the 1960’s, [15, 16]. In our cohort, 9 infants received conservative treatment, and 26 infants underwent surgical intervention. The overall mortality rate was 8.6%, which was similar to that reported in the literature [1, 13]. Patients with only a small amount of ascites and mild distention of the bowel loops may not need surgery, according to our current study.

## Conclusions

In conclusion, our current study demonstrated that fetal MRI could help diagnose MP with various findings, including abnormal fetal ascites, distended bowel loops, hydroceles, micro-colorectum, bowel dilatation, peritoneal calcifications and meconium pseudocysts. Particularly, the existence of micro-colorectum and/or bowel dilatation reflected the need for postnatal surgical intervention.

## Data Availability

The dataset analyzed during the current study is available from the corresponding author on reasonable request pending the approval of our institution and trial/study investigators who contributed to the dataset.

## Abbreviations

MP: meconium peritonitis
MRI: magnetic resonance imaging
SPAIR: spectral presaturation attenuated inversion recovery echo sequences
TR: time of repetition
TE: time of echo
HBV: hepatitis B virus
HASTE: Half-Fourier acquisition single-shot turbo spin-echo
true FISP: true fast imaging with steady-state precession

## References

[1] Nam SH, Kim SC, Kim DY, Kim AR, Kim KS, Pi SY (2007). Experience with meconium peritonitis. J Pediatr Surg.

[2] Martínez Ibáez V, Boix-Ochoa J, Roca JL, Ruiz H. Meconial peritonitis: conclusions based on 53 cases. Cirugía pediátrica: organo oficial de la Sociedad Espaola de Cirugía Pediátrica. 1900;3(2):80–2. 2252854

[3] Foster MA, Nyberg DA, Mahony BS, Mack LA, Marks WM, Raabe RD (1987). Meconium peritonitis: prenatal sonographic findings and their clinical significance. Radiology..

[4] Tibboel D, Gaillard JL, Molenaar JC (1986). The importance of mesenteric vascular insufficiency in meconium peritonitis. Hum Pathol.

[5] Regev RH, Markovich O, Arnon S, Bauer S, Dolfin T, Litmanovitz I (2009). Meconium periorchitis: intrauterine diagnosis and neonatal outcome: case reports and review of the literature. J Perinatol.

[6] Shyu MK, Shih JC, Lee CN, Hwa HL, Chow SN, Hsieh FJ (2003). Correlation of prenatal ultrasound and postnatal outcome in meconium peritonitis. Fetal Diagn Ther.

[7] Frates MC, Kumar AJ, Benson CB, Ward VL, Tempany CM (2004). Fetal anomalies: comparison of MR imaging and US for diagnosis. Radiology..

[8] He F, Yin Y, Huang L, Li H, Cao Y (2018). Using prenatal MRI to define features of meconium peritonitis: an overall outcome. Clin Radiol.

[9] Simpson JY (1838). Contributions to intra-uterine pathology. Part I. notices of cases of peritonitis in the Foetus in utero. Edinb Med Surg J.

[10] Chan KL, Tang MH, Tse HY, Tang RY, Tam PK (2005). Meconium peritonitis: prenatal diagnosis, postnatal management and outcome. Prenat Diagn.

[11] Smith FW (1983). NMR imaging in pediatric practice. Pediatrics..

[12] Ertl-Wagner B, Lienemann A, Strauss A, Reiser MF (2002). Fetal magnetic resonance imaging: indications, technique, anatomical considerations and a review of fetal abnormalities. Eur Radiol.

[13] Ping LM, Rajadurai VS, Saffari SE, Chandran S (2017). Meconium peritonitis: correlation of antenatal diagnosis and postnatal outcome - an institutional experience over 10 years. Fetal Diagn Ther.

[14] Nyberg DA, Mack LA, Patten RM, Cyr DR (1987). Fetal bowel. Normal sonographic findings. J Ultrasound Med.

[15] Wang CN, Chang SD, Chao AS, Wang TH, Tseng LH, Chang YL (2008). Meconium peritonitis in utero---the value of prenatal diagnosis in determining neonatal outcome. Taiwan J Obstet Gynecol.

[16] Zangheri G, Andreani M, Ciriello E, Urban G, Incerti M, Vergani P (2007). Fetal intra-abdominal calcifications from meconium peritonitis: sonographic predictors of postnatal surgery. Prenat Diagn.

